# Differential regional importance mapping for thyroid nodule malignancy prediction with potential to improve needle aspiration biopsy sampling reliability

**DOI:** 10.3389/fonc.2023.1136922

**Published:** 2023-04-28

**Authors:** Liping Wang, Yuan Wang, Wenliang Lu, Dong Xu, Jincao Yao, Lijing Wang, Lei Xu

**Affiliations:** ^1^ Department of Ultrasonography, The Cancer Hospital of the University of Chinese Academy of Sciences (Zhejiang Cancer Hospital), Institute of Basic Medicine and Cancer, Chinese Academy of Sciences, Hangzhou, China; ^2^ School of Mathematical Sciences, Zhejiang University, Hangzhou, China; ^3^ Department of Ultrasound, Zhejiang Society for Mathematical Medicine, Hangzhou, China; ^4^ Group of Computational Imaging and Digital Medicine, Zhejiang Qiushi Institute for Mathematical Medicine, Hangzhou, China

**Keywords:** tumor heterogeneity, ultrasound guided biopsy, class activation map, artificial intelligence, thyroid nodule

## Abstract

**Objective:**

Existing guidelines for ultrasound-guided fine-needle aspiration biopsy lack specifications on sampling sites, but the number of biopsies improves diagnostic reliability. We propose the use of class activation maps (CAMs) and our modified malignancy-specific heat maps that locate important deep representations of thyroid nodules for class predictions.

**Methods:**

We applied adversarial noise perturbations to the segmented concentric “hot” nodular regions of equal sizes to differentiate regional importance for the malignancy diagnostic performances of an accurate ultrasound-based artificial intelligence computer-aided diagnosis (AI-CADx) system using 2,602 retrospectively collected thyroid nodules with known histopathological diagnosis.

**Results:**

The AI system demonstrated high diagnostic performance with an area under the curve (AUC) value of 0.9302 and good nodule identification capability with a median dice coefficient >0.9 when compared to radiologists’ segmentations. Experiments confirmed that the CAM-based heat maps reflect the differentiable importance of different nodular regions for an AI-CADx system to make its predictions. No less importantly, the hot regions in malignancy heat maps of ultrasound images in comparison with the inactivated regions of the same 100 malignant nodules randomly selected from the dataset had higher summed frequency-weighted feature scores of 6.04 versus 4.96 rated by radiologists with more than 15 years of ultrasound examination experience according to widely used ultrasound-based risk stratification American College of Radiology (ACR) Thyroid Imaging Reporting and Data System (TI-RADS) in terms of nodule composition, echogenicity, and echogenic foci, excluding shape and margin attributes, which could only be evaluated on the whole rather than on the sub-nodular component levels. In addition, we show examples demonstrating good spatial correspondence of highlighted regions of malignancy heat map to malignant tumor cell-rich regions in hematoxylin and eosin-stained histopathological images.

**Conclusion:**

Our proposed CAM-based ultrasonographic malignancy heat map provides quantitative visualization of malignancy heterogeneity within a tumor, and it is of clinical interest to investigate in the future its usefulness to improve fine-needle aspiration biopsy (FNAB) sampling reliability by targeting potentially more suspicious sub-nodular regions.

## Introduction

Thyroid nodules are detected in as high as 65% of the population (1), and thyroid cancer is one of the most frequently occurring malignant tumors. Meanwhile, ultrasound imaging is the most commonly used method for evaluating thyroid nodules, given its ease to detect the nodules and good sensitivity to differentiate benign from malignant tumors as well as its non-invasive nature with widespread accessibility in clinics. However, the diagnosis of thyroid nodules is highly dependent on radiologists’ personal experience and subjective judgment, leading to not uncommonly inconsistent conclusions. Currently, cytopathological examination performed on minimally invasive fine-needle aspiration biopsy (FNAB) typically has a diagnostic sensitivity and specificity that vary at 65%–98% and 72%–100%, respectively (2–4). More importantly, as cancerous masses are typically heterogeneous (5, 6), it is of clinical importance to be able to differentiate regions of different malignancy levels within the same imaged tissue such that FNAB sampling can be more precisely guided. To date, there are numerous practical guidelines about under what circumstances FNAB shall be applied (7–9), but there exists no consensus or guideline about the number of needle-sampling passes to acquire adequate specimens for diagnostic purposes, let alone recommendations for precise sampling sites in nodules under ultrasound guidance. With the advancement of artificially intelligent technologies and especially the development of deep learning algorithms, it becomes increasingly common for radiologists to include these auxiliary mathematical models in their toolboxes during clinical studies for disease detection and diagnosis (10–12).

Surprisingly or not, the capability of convolutional neural networks (CNNs) to approximate any arbitrary functions has become a double-edged sword, as any insights about how the models come to their conclusions are hardly accessible to human understanding given their architectural designs. Some proposed circumventing strategies include weighting the predicted classification probability of a CNN model as a contributing factor together with other human-interpretable image features (13) and taking the similarities between imaged lesions with known diagnosis as an extra channel of information to guide human-centered diagnosis (14).

Other possibilities to retrieve hints using computer-aided artificial intelligence diagnosis as we perceive include generating the class activation map (CAM) (15) that localizes the deep representation of class-discriminating image regions. In the field of medical imaging, CAMs have been employed to visualize hot regions that conclude each predicted classification type of tissue (16–18) using a heat map representation. To date, it is however mostly a visualization tool and lacks quantitative validation to show whether hot regions in such heat maps for malignant samples indeed possess higher importance in determining the classification type. If so, the question of how much more important the hot regions are relative to other regions still awaits an answer.

To answer these questions, we designed this proof-of-concept computational study described as the following. We first generated CAMs in two different rendering configurations using the Software Development Kit (SDK) of the artificial intelligence computer-aided diagnosis (AI-CADx) system referred to as “AI-SONIC™ Thyroid” for thyroid nodule diagnoses. In the first configuration, the heat maps were rendered conventionally such that they make no distinctions between benign and malignant cases visually, and the color temperature is supposed to show the associated regional importance in predicting the classification regardless of what the predicted type is. In the second configuration, however, the intensities of these heat maps were normalized to the malignancy probabilities predicted by the AI-CADx system and rendered in such a way that the more reddish the color, the higher probability that the nodule is predicted to be malignant, whereas the bluish color indicates benignity. In other words, the second visualization configuration presents essentially malignancy heat maps.

To quantitatively analyze the importance of different regions within the ultrasound-imaged thyroid nodules in the diagnosis by the AI-CADx system, we subdivided each nodular CAM into five concentric areas of the same size and then evaluated the AI diagnostic performances after the adversarial noises (19) were applied to each subdivided nodular region in ultrasound images. The motivation behind this is that, hypothetically, the regions of higher importance for predicting the correct diagnosis of thyroid nodules should be more vulnerable to noise perturbations. As a second attempt, we also performed a test by varying the heat intensity threshold to segment CAMs and evaluated how the diagnoses were based on regions above the thresholds.

## Materials and methods

### Data acquisition

The ultrasound images covering a total of 2,602 thyroid nodules from 2,488 patients were collected between January 2011 and February 2019 by The Cancer Hospital of the University of Chinese Academy of Sciences in Hangzhou, Zhejiang, China. All nodules were diagnosed by surgical pathological examinations, among which 1,581 cases were determined to be benign and 1,021 cases were found to be malignant. The local ethics committee waived the ethical approval in view of the retrospective nature of the study, and all reviews of the ultrasound image and postsurgical hematoxylin and eosin (H&E)-stained pathological images being performed were part of the clinical routine.

### AI-CADx model

This study was based on the AI-SONIC™ Thyroid nodule diagnosis system (Demetics Medical Technology, Hangzhou, China). It is built on the EfficientNet (20) architectural backbone and employs supervised sharpness-aware minimization for model parameter optimization to realize automatic nodule segmentation and classification (21). The classification module of the AI model relies on the precise localization of the nodules. This system can automatically detect thyroid nodules in two-dimensional grayscale ultrasound images and output corresponding masks. Therefore, radiologists do not need to manually outline the thyroid nodules except on rare occasions when manual corrections are necessary.

### Heat map

All the generated heat maps in this study were based on CAM (15) using global average pooling (GAP) in CNNs. Before the final output layer for image classification, we performed global average pooling on the convolutional feature maps and used those as features for a fully connected layer that produces the desired output. We projected back the weights of the output layer onto the convolutional feature maps using the cited CAM method. Global average pooling outputs the spatial average of the feature map of each unit at the last convolutional layer. A weighted sum of these values is used to generate the final output. We computed a weighted sum of the feature maps of the last convolutional layer to obtain our class activation maps.

For an image, we used $g_{k}(x,y)$ to represent the activation of unit k in the last convolutional layer at the spatial location. Next, for unit (x,y) , G^k^ is the result of performing global average pooling, which is defined as $\sum_{x,y}g_{k}\left(x,y\right)$ . Then, for a class i, the input to the softmax S_i_ is $\sum_{k}\omega_{k}^{i}G_{k}$ , where the $\omega_{k}^{i}$ is weight corresponding to class i for unit k. In particular, the $\omega_{k}^{i}$ implies the importance of class i. The final output of the softmax for class i, Pi is given by $\frac{\exp(S_{i})}{\sum_{i}\exp(S_{i})}$ . The bias term was ignored as in the original paper by setting the input bias of the softmax to 0 to have no impact on the classification performance.

By adding ${\text{G}}_{k}=\sum_{\text{x},\text{y}}{\text{g}}_{k}\left(\text{x},\text{y}\right)$ into the class score, we have the following:


$$\begin{array}{l} S_{i}=\sum_{k}\omega_{k}^{i}\sum_{x,y}g_{k}\left(x,y\right)\\ \;=\sum_{x,y}\sum_{k}\omega_{k}^{i}g_{k}\left(x,y\right) \end{array}$$

In addition, we define Mi as the class activation map for class i, where each spatial element is the product of the weight $\omega_{k}^{i}$ and the activation $g_{k}(x,y)$ given by the following:


$$M_{i}\left(x,y\right)=\sum_{k}\omega_{k}^{i}g_{k}\left(x,y\right)$$

Therefore, $S_{i}=\sum_{x,y}M_{i}\left(x,y\right)$ and $M_{i}(x,y)$ directly show the importance of the activation at spatial grid (x, y) leading to the classification of an image to class i.

The CAM is a weighted linear sum of the presence of these visual patterns at different spatial locations. By upsampling the class activation map to the size of the input image, we could identify the image regions most relevant to the particular category.

### Nodular region segmentation and noise perturbation

We divided each CAM of thyroid nodules into five nearly concentric regions of equal sizes. This was performed first by setting a binarization threshold to 0 to obtain a nodular segmentation with a total area of N. Then, we searched for a second threshold for level setting the CAM to segment the outermost region with the size of N/5. We iterated this process until we segmented out the innermost region with the same size.

To illustrate the different importance of each region of the heat map, we added perturbations to the different regions. A commonly employed first-order adversarial attack method—Fast Gradient Sign Method (FGSM) (19)—was used to generate perturbed thyroid ultrasound images.

Let x be the input to the model, y the output to the model (the targets associated with x), *ϕ* the parameters of a model, and *L*(*x*, *y*, *ϕ*) the cost function used to train the neural network. Then, we can linearize the cost function about the current value of *ϕ*, acquiring an optimal max-norm constrained perturbation of


$$\delta =\beta sign\left(\nabla_{x}L(x,y,\varphi )\right)$$

where *β* is a predefined perturbation size, which represents the maximum change to pixel values of an image. sign() is a symbolic function, which is defined as


$$sign(x)=\left\{ \begin{array}{c} 1,\;x>0\\ 0,\;x=0\\ -1,\;x<0 \end{array}\right.$$

In addition, we used the back-propagation to compute the required gradient.

This method can reliably generate perturbations to the input of the model. The single-step FGSM perturbs the original example by a fixed amount along the direction (sign) of the gradient of loss function such that the result from the perturbed image is given by the following:


$$x^{\prime }=x+\beta sign(\nabla_{x}L(x,y,\varphi ))$$

### Statistical methods

To assess the performance of the AI-CADx system, we computed the receiver operating characteristic (ROC) curve and used the area under the curve (AUC) as the evaluation metric. For each nodule, we randomized five times the positions in segmented nodular regions where perturbation noises were applied, and we used the malignancy probabilities averaged over five times of randomized noise perturbations to compute the ROC curves and the subsequent AUC values. To compare individual ROC curves, we performed DeLong’s test (22) to evaluate whether the differences in AUC values were statistically significant.

For further statistical significance validation, we evaluated AUC values computed for different regions in each dataset (the division of which is described above), followed by paired t-test for different segmented nodular regions.

### Ultrasound feature evaluations in highlighted regions versus the inactivated regions

We randomly selected 100 malignant nodules from our dataset and had the ultrasound features rated by radiologists with 15 years of ultrasound examination experience according to widely used ultrasound-based risk stratification American College of Radiology (ACR) Thyroid Imaging Reporting and Data System (TI-RADS) (23) in terms of nodule composition, echogenicity, and echogenic foci. We excluded feature evaluation based on shape and margin attributes, as they, by design, could only be evaluated on the whole rather than on the sub-nodular component levels. We included the statistical evaluation of sub-component localizations for the highlighted and inactivated regions visualized through our proposed malignancy heat map representation, however without any associated risk scores. Each scored feature was weighted against its occurrence frequency and multiplied with the risk points given by ACR TI-RADS criteria to gain an overview of the average ultrasound feature profile of the highlighted regions in contrast to the inactivated regions of our proposed malignancy heat map.

### Histopathological correspondence evaluation of malignancy heat map

H&E staining images of postoperative histopathological slides with saved ultrasound images of matched sectional views were reexamined by a senior pathologist with 25 years of work experience with the boundaries and shapes of the malignant regions of thyroid tumors on the H&E slides outlined using software provided by digital pathology slide scanner KF-PRO-005-EX (Konfoong Biotech International Co., Ltd., Yuyao, China).

## Results

### Heat maps for benign and malignant nodule classifications

**Figure 1:**
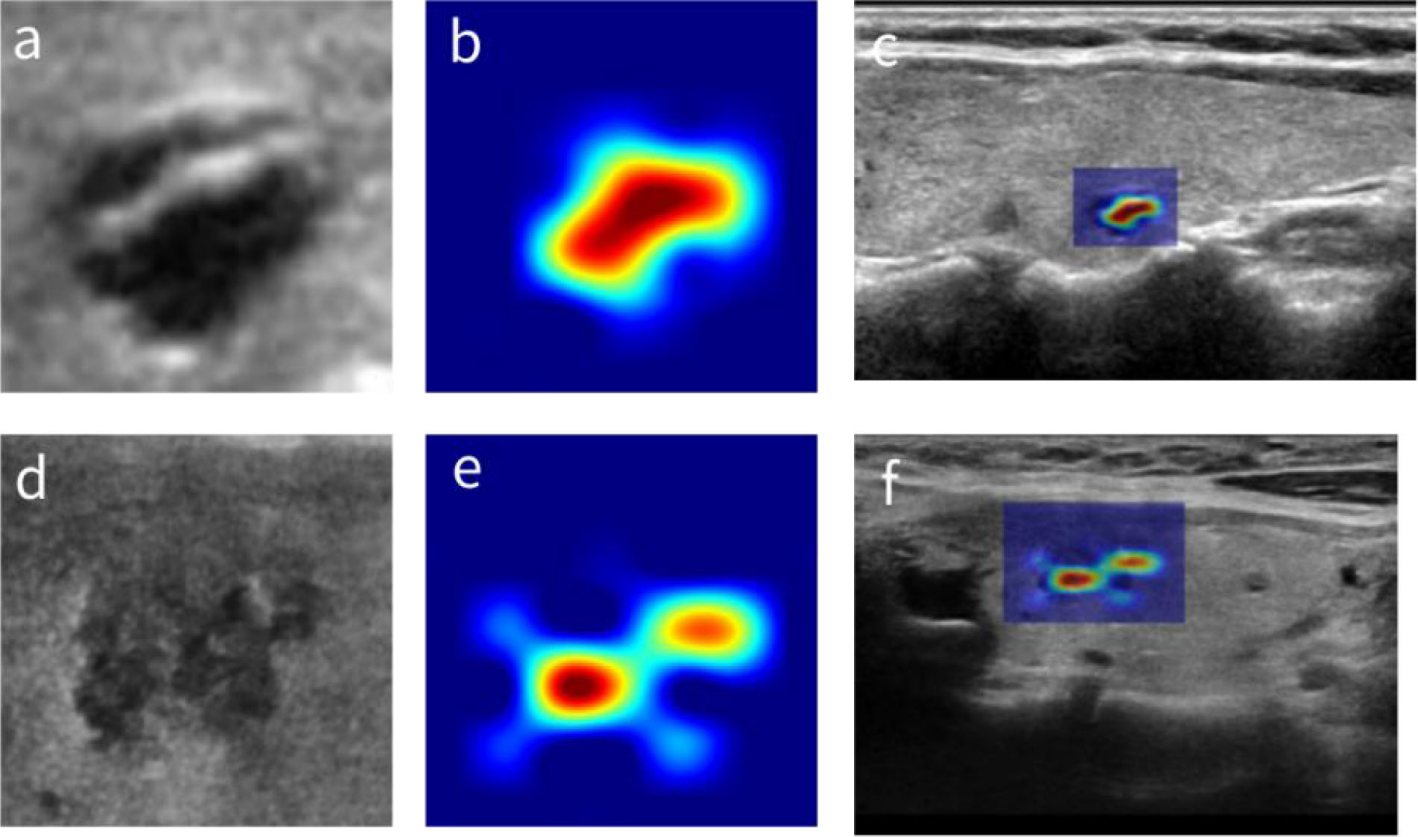
The CAMs of thyroid nodules. **(A)** A benign thyroid nodule. **(B)** The corresponding CAM. **(C)** The superposition of panels **(A, B)** in the original image. **(D)** A malignant thyroid nodule. **(E)** The corresponding CAM. **(F)** The superposition of panels **(D, E)** in the original image. CAMs, class activation maps.

Representative examples of the CAMs generated by using AI-SONIC™ Thyroid SDK are shown in **Figure 1**. The first row shows a benign thyroid nodule, and the second row shows a malignant thyroid nodule. The maps highlight the discriminative image regions for thyroid nodule classification. On the left column, zoomed-in thyroid nodule images are shown, whereas the middle column shows the corresponding CAMs. The thyroid images together with their CAMs are superimposed and shown in the original images on the right column. We can see from the exemplars that the malignant nodule has a more complicated CAM profile than the benign one, which may reflect some correlation with the shapes of nodular margins.

### Influence of noise perturbations to different CAM regions on thyroid nodule diagnosis

**Figure 2:**
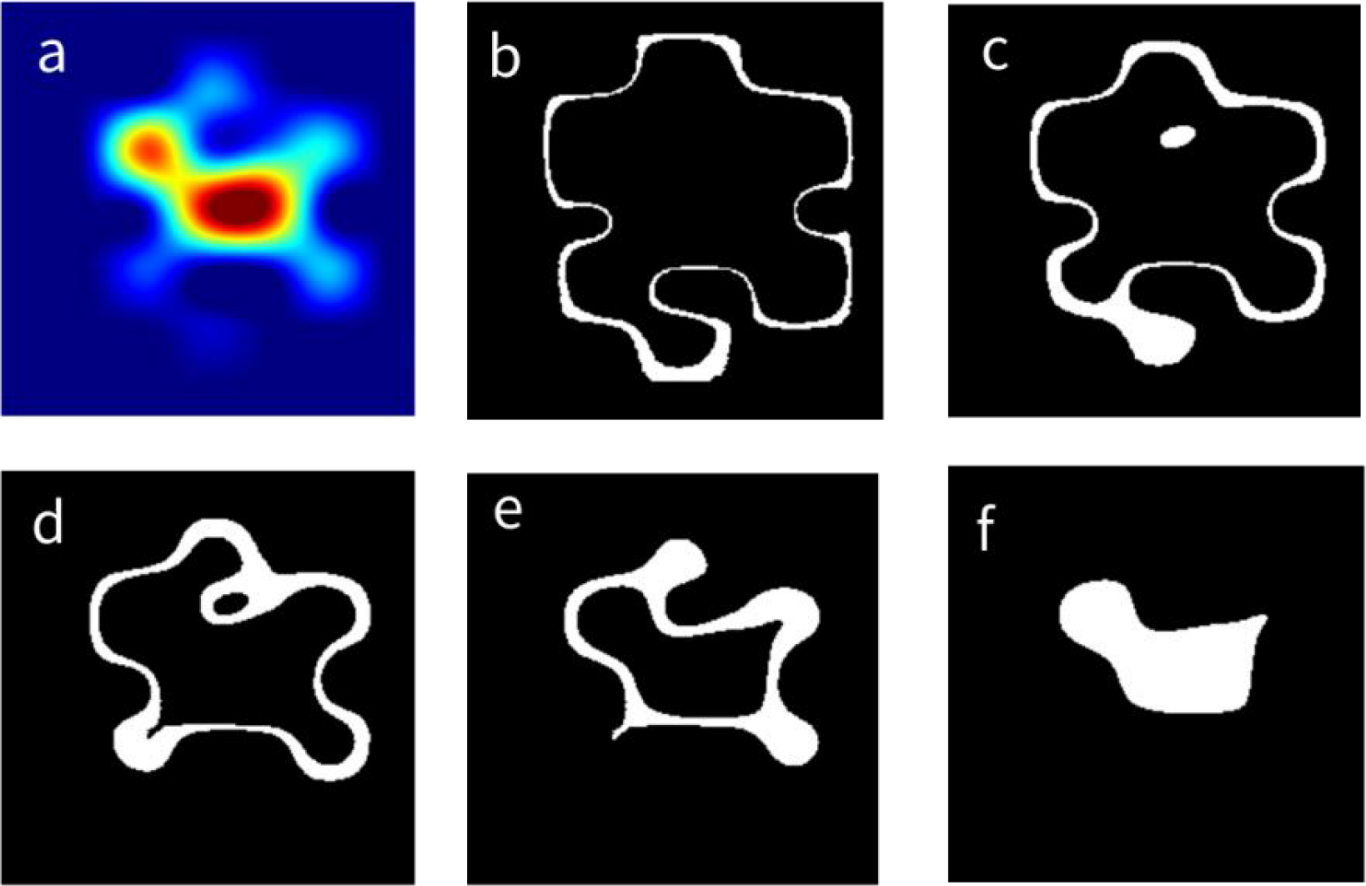
Division of a CAM into five equal parts. **(A)** The CAM of a thyroid nodule. **(B–F)** The segmented region from the outermost region (labeled as region I) to the innermost region (labeled as region V), respectively. CAM, class activation map.

In order to evaluate whether different regions of thyroid nodules may contribute differently to their classifications by the AI-CADx system, we first segmented individual CAMs into five concentric areas of equal sizes according to the method section about nodular CAM segmentation as described above. An illustration of how the segmentation of a nodular CAM looks is given in **Figure 2**.

**Figure 3:**
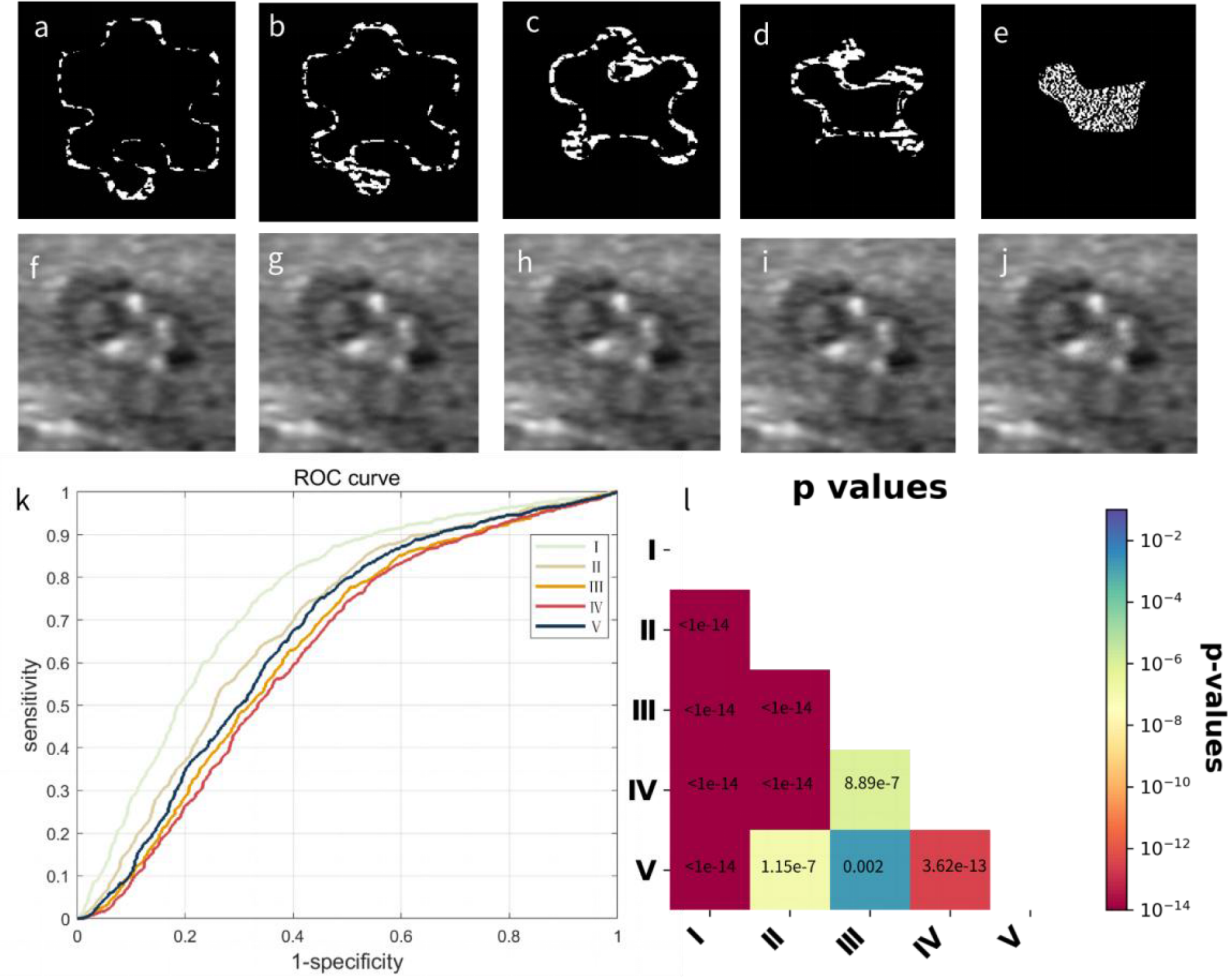
Adding noise perturbations to five regions from the outermost area to the innermost area of equal sizes in the original image and their influence on classification performances. **(A–E)** The generated gradient sign noise images. **(F–J)** The perturbed images with noises with magnitude *β* of 0.0136 (identified as the point where the overall AUC values decay the fastest as measured by absolute gradient) added to each corresponding region. **(K)** ROC curves calculated from the complete dataset with nodular regions separately perturbed by noises in regions I to V represent the outermost to innermost nodular regions. **(L)** The associated p-value matrix for statistical comparisons. All p-values were ≤0.002, and p-values lower than 1 × 10^−14^ were shown as 1 × 10^−14^. Self-comparisons were omitted, as they were constant at 1. AUC, area under the curve; ROC, receiver operating characteristic.

We then added gradient sign noise perturbation, which is commonly used for adversarial attacks for CNN models, to each segmented region in the original ultrasound images as shown in **Figure 3**. The first row shows the noise images added to each segmented region (**Figure 2**), and the second row shows the noise-perturbed images. The noises added to the original images are barely visible to human eyes but do have a strong impact on the diagnostic performance of the AI-CADx system. We chose the noise magnitude *β* to be 0.0136 by searching for the maximum absolute gradient of AUC values with respect to the noise magnitudes (**Supplementary Figure 1**). As a control, the ROC curve and the corresponding AUC value calculated for non-perturbed images are given in **Supplementary Figure 2**.

All thyroid nodule images with noises added to different regions were then classified, and the ROC curves were computed for different noise-perturbed regions, as shown in **Figure 3K**. To statistically compare the influence of noise perturbation on different regions using ROC curves, we computed pairwise p-values according to DeLong’s test (16), with the results shown in **Figure 3K**. Note that we skipped the statistical comparisons against oneself, as in this case, the p-values were constant 1. It can be seen that it was not the innermost or hottest region (green line for region I) from the CAMs that were worst affected by noises but region IV (purple line) followed by region III (orange line), suggesting that the tissues surrounding the core area identified by CAMs played a crucial role in the benign and malignant nodule diagnoses.

To further validate this observation, we subdivided the complete dataset into five subsets randomly, summarized in **Table 1**.

**Table 1:** The subdivided datasets for subsequent nodular region comparison experiment.

Dataset	Total nodules	Benign	Malignant
1	520	320	200
2	520	329	191
3	520	300	220
4	520	322	198
5	522	310	212

**Figure 4:**
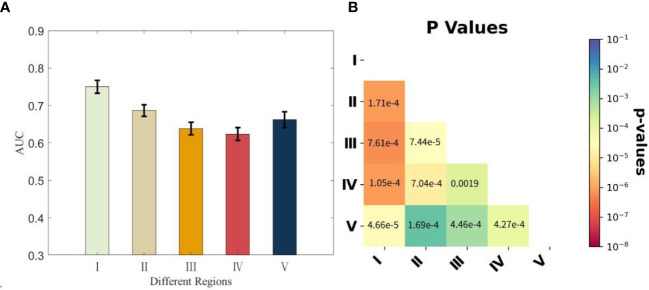
The AUC values calculated from the five subdivided datasets with nodular regions separately perturbed by noises and the associated p-value matrix for statistical comparisons. **(A)** Each bar representing the corresponding region is presented with the average AUC values over the five subdivided datasets and the standard deviations. **(B)** All p-values were<0.004, and self-comparisons were omitted, as they were constant at 1. AUC, area under the curve.

We calculated the AUC values for each dataset in which noise perturbations were applied in the same way as described above to individually segmented nodular regions, and we computed their average values and standard deviations over the five datasets as well as the p-value matrix for pairwise comparisons. The corresponding results are given in **Figure 4**.

### Influence of noise perturbations to different malignancy regions on thyroid nodule diagnosis

As cases with suspicious malignant features are more clinically important to be identified in order for subsequent treatment planning, for instance, FNAB, to verify the malignancy status, we modified the conventional CAMs by multiplying the malignancy probability predicted by the AI-CADx model with the CAM specific for cases suspicious for being malignant to generate malignancy heat maps.

**Figure 5:**
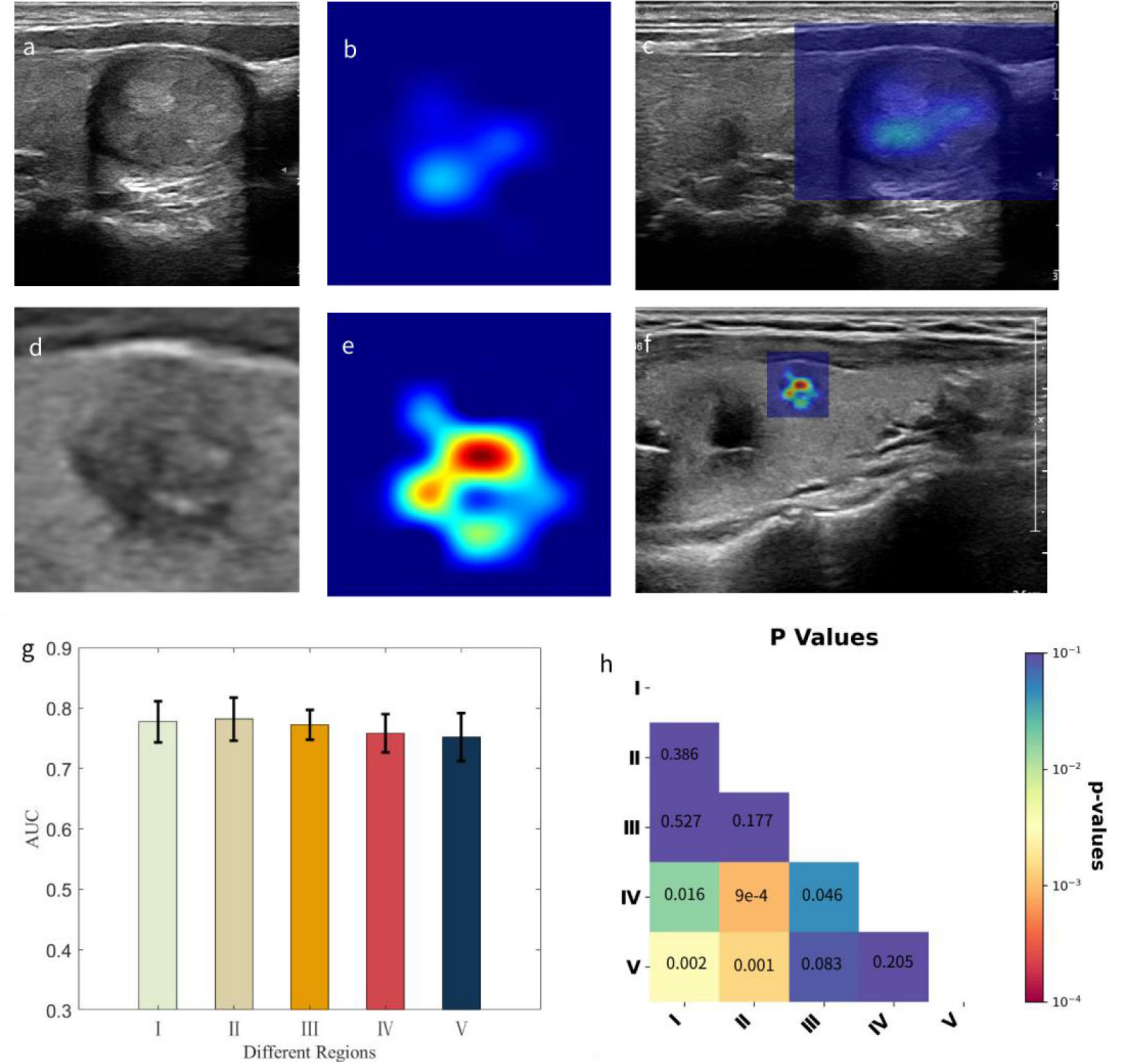
Our proposed malignancy heat map and the influence of noise perturbations to five different regions on classification performances. **(A, D)** The cropped zoomed-in images for benign and malignant nodules, respectively. **(B, E)** The corresponding heat maps with color temperature bounded by the predicted malignant probabilities (0.2907 *vs.* 0.9382) by the AI-CADx system. **(C, F)** The superimposed images for the corresponding nodules and heat maps. **(G)** Each bar representing the corresponding region is presented with the average AUC values over the five subdivided malignant datasets and the standard deviations. **(H)** The associated p-value matrix for statistical comparisons. The smallest p-values were<0.001, and self-comparisons were omitted, as they were constant at 1. AI-CADx, artificial intelligence computer-aided diagnosis; AUC, area under the curve.

We performed nodular segmentation and applied noise perturbations in the same way as described previously in the Materials and Methods section. In this experiment, we also randomly subset the samples with predicted malignancy scores of higher than 0.4 (below which the probability of being malignant was approximately 3%) to five datasets for the purpose of ensuring reproducibility. The results together with the p-value matrix for pairwise comparisons are summarized in **Figure 5**. It can be found that in this case, adding noises to innermost region V has the greatest influence on thyroid nodule diagnostic performances, correlating well with the heat intensity profile (**Figure 5**).

### Correlation of highlighted regions with higher risks versus inactivated regions of the heat maps

To investigate whether the highlighted regions had a higher correlation with malignancy risks compared to the inactivated regions of our proposed malignancy heat map, radiologists with more than 15 years of ultrasound examination experience evaluated the ultrasound features according to the ACR TI-RADS risk stratification criteria in terms of nodular composition, echogenicity, and echogenic foci, which are applicable to subcomponent evaluations. The results summarized from 100 randomly selected malignant nodules in **Table 2** show that the more centrally localized highlighted regions in the malignancy heat maps had higher summed weighted risk scores of 6.04 when compared to the inactivated regions of 4.96, demonstrating a higher correlation with the malignancy risks. On the whole, the highlighted regions turn out to be more hypoechoic and more likely to contain punctate calcifications.

**Table 2:** The ACR TI-RADS feature analysis of CAM highlighted and inactivated regions in 100 malignant nodules randomly selected from the dataset.

Features	CAM hot regions (100)			CAM inactivated regions in the same nodules (100)		
Localization	Frequency	Probability	Weighted score	Frequency	Probability	Weighted score
On the margin	27	0.27	–	93	0.93	–
Not on the margin	73	0.73	–	7	0.07	–
Echogenicity						
Very hypoechoic	84	0.84	2.52	43	0.43	1.29
Hypoechoic	16	0.16	0.32	52	0.52	1.04
Isoechoic	0	0.00	0	5	0.05	0.05
Composition						
Solid	100	1.00	2	100	1.00	2
Echogenic foci						
Macro-calcification	0	0.00	0	16	0.16	0.16
Punctate calcification	40	0.40	1.2	14	0.14	0.42
None	60	0.60	0	70	0.70	0
**Sum**	100	1	6.04	100	1	4.96

Note that the nodular shapes and margin features in ACR TI-RADS are defined for the whole nodules but not sub-nodular regions. Therefore, only relative localizations are provided, which are however not associated with defined risk points.

ACR, American College of Radiology; TI-RADS, Thyroid Imaging, Reporting and Data System; CAM, class activation map.

### Pathological significance of the heat maps

**Figure 6:**
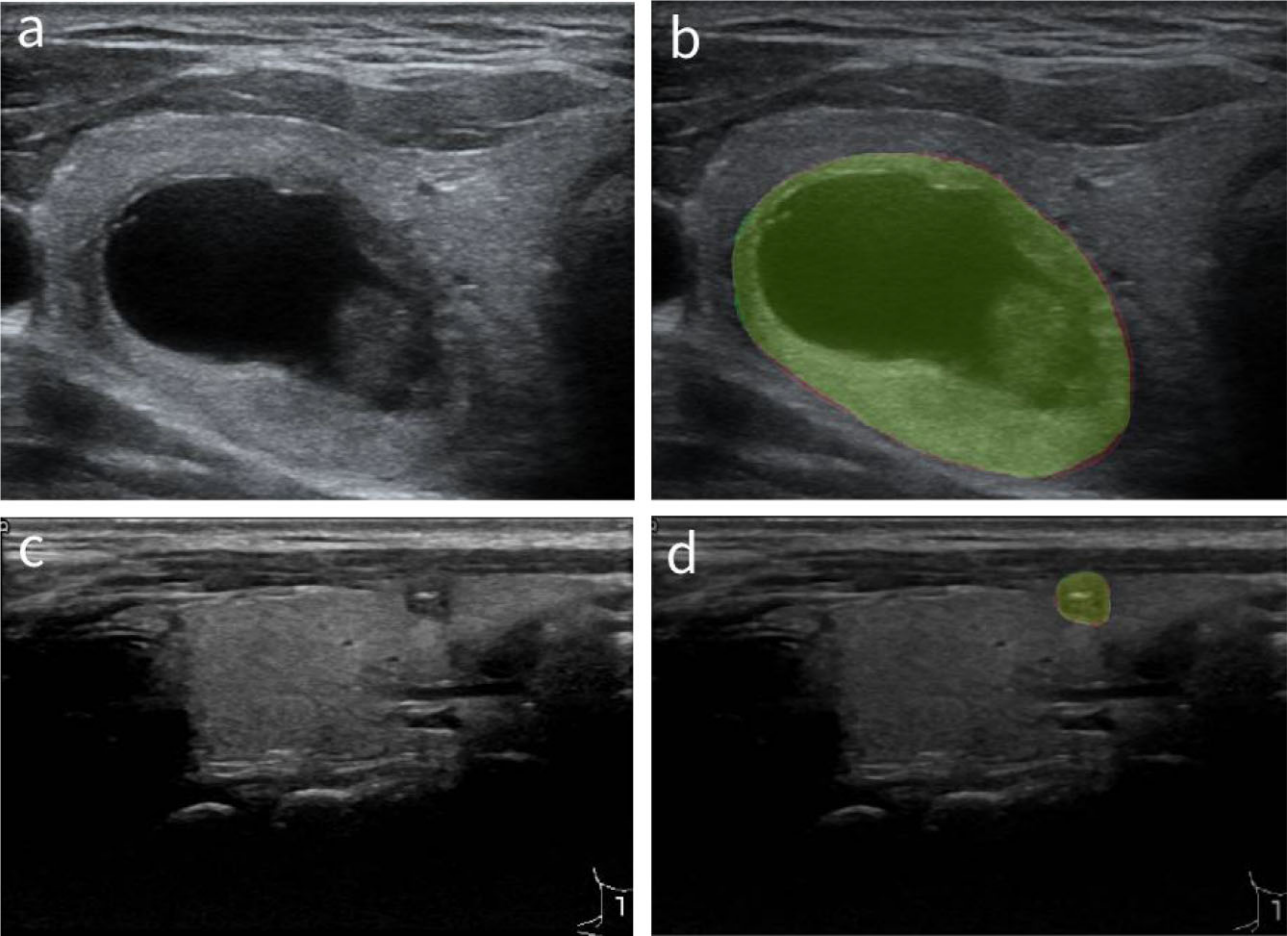
Comparison of the masks generated by the AI system and the masks outlined by the radiologists. **(A, C)** The original thyroid ultrasound images. **(B, D)** Masks generated by the AI system and radiologists, where the red segments are produced by the AI system, and the green ones are manually drawn by the radiologists. AI, artificial intelligence.

**Figure 7:**
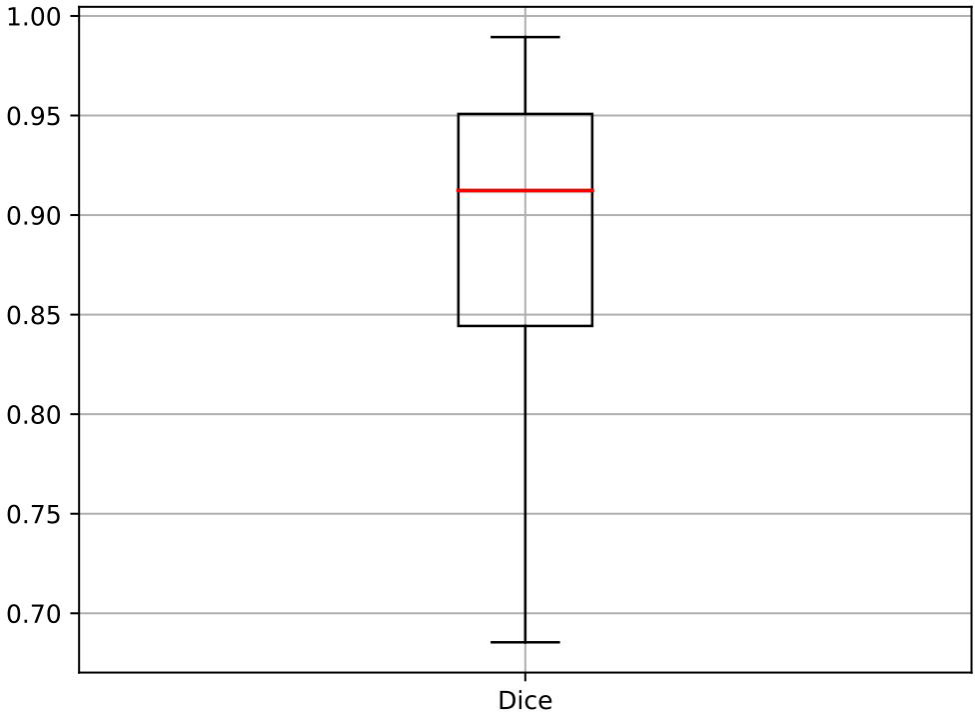
The boxplot of the Dice coefficients for quantitative evaluation of the automatically segmented nodules by the AI system in comparison to radiologists’ segmentations. AI, artificial intelligence.

In order to verify the pathological significance of the heat maps, we first compared the nodule masks generated by the AI system with the masks outlined by the radiologists of different nodule sizes in **Figure 6** to show that the basis for computing the heat maps is pathologically relevant. We calculated the dice similarity coefficient (Dice) as a metric to evaluate how well the AI system performs for localizing thyroid nodules within the gland tissues, with the result shown by the boxplot in **Figure 7**. It can be found that the masks outputted by the AI system are highly overlapped with the masks delineated by the radiologists (the median dice coefficient >0.9), demonstrating the high accuracy of the AI system in identifying the nodule areas. This verifies that the area of the heat map is mainly in the lesion area, as nodule segmentation is a key step for heat map computations.

**Figure 8:**
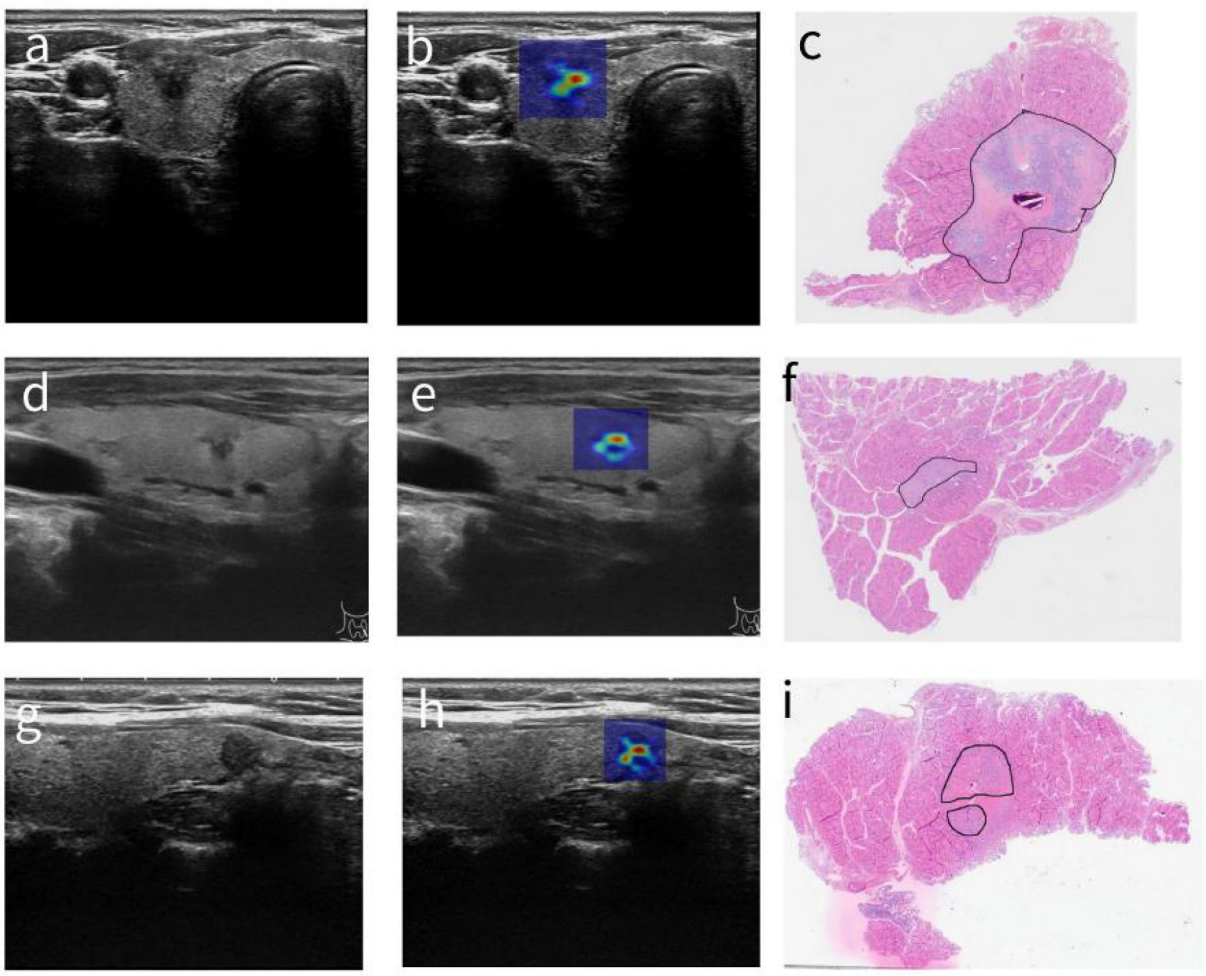
Correspondence of post-operation pathological images and malignancy heat maps computed on ultrasound images. **(A, D, G)** The original thyroid ultrasound images. **(B, E, H)** The superimposed images for the corresponding nodules and heat maps. **(C, F, I)** The cropped zoomed-in pathological images.

To further demonstrate and qualitatively verify the pathological relevance of the computed heat maps, we compared the post-operation pathological images with heat maps computed on ultrasound images shown in **Figure 8**. It shows that the shapes of cancer cell-rich areas in pathological images outlined by the pathologists have very good correspondence to “hot regions” in our proposed malignancy heat maps, suggesting that the malignancy heat map has promising potential to provide accurate sampling position guidance for FNAB. It is interesting to observe that in **Figure 8G** where there is clearly one single nodule in the original ultrasound image, the malignancy heat map shows two strongly activated subregions that correspond nicely with the two malignant cell-enriched regions outlined in H&E-stained histopathology images.

## Discussion

In this study, we proposed to take advantage of CAMs for differentiating regions in the ultrasound images of thyroid nodules that may contribute differently to the diagnosis by an AI-CADx system. We segmented the CAMs of individual nodule images into five concentric areas of equal sizes, applied adversarial noise perturbations to the different segmented regions, and evaluated the impact on the diagnostic performances of the AI-CADx system. Our results confirmed that the CAMs can reflect the importance of different degrees in different nodular regions for a CNN-based diagnosis system to make its predictions according to our noise perturbation experiments. Surprisingly or not, it was found that it was not the innermost region or in other words the “hottest” region seen in the CAMs that were most severely influenced by the noise perturbations as one might presumably expect. This phenomenon was again observed in experiments where we randomly divided the original dataset into five subsets and verified by the statistical significance tests, suggesting that the regional sensitivities to noise perturbations may not perfectly correlate with the heat intensity profile in conventional CAM for nodular malignancy state predictions.

We then tried modifying the conventional CAM by multiplying the heat maps generated by CAM with the predicted malignancy probabilities by the AI-CADx system to produce malignancy heat maps. The resulting heat maps can therefore visually convey information about the malignancy predictions for individual nodules. Furthermore, this malignant nodule-specific modification to the conventional CAM would in principle allow more insightful inspection of regional importance for malignancy assessment and thus provide guidance to doctors about where the FNAB should be mostly directed to. In this case, the innermost nodular area defined by the heat map was found to be the most sensitive region to noise perturbations, which could be due to the multiplication of malignancy probability, which improves its malignancy relevance and subsequently the perturbation responsiveness, though it was not statistically different from the region adjacent to it, which suggests that both regions are the most critical for determining malignant thyroid nodule diagnosis.

By comparing the influences of noises on AI-CADx performance for thyroid nodule diagnosis by perturbing regions segmented according to the conventional CAMs that analyze both benign and malignant nodules and our proposed variant that concentrates on suspicious nodules, it can be seen that noise perturbations to inner regions defined in CAMs had a more dramatic effect on datasets not excluding benign nodules predicted with high confidence. This could be because the inner regions of benign nodules are more vulnerable to noise perturbations, while the malignant nodules are more resistant to noise perturbations. This can also explain why the regional differences in suspicious malignant nodules were less abrupt. Nonetheless, the central region defined by the malignancy heat map was significantly more sensitive to noise perturbation, suggesting the potential of this technique for differentiating tumor heterogeneity.

As an alternative to the noise perturbation experiments, we have also tried varying binary thresholds to segment the CAMs and evaluated the diagnostic performance of the AI-CADx system directly on the ultrasound images of the regions above the thresholds with the hope of finding an appropriate heat intensity threshold that could permit a satisfactory cutoff to differentiate insignificant regions from regions where special attention would be paid to. However, such a conclusion could not be drawn from this attempt, as the AUC values (**Supplementary Figure 3**) showed a continual decrease with the heat intensity threshold. This can be attributed to the fact that the marginal shapes of thyroid nodules are crucial for malignancy diagnosis because the segmented regions can have very complicated margins and even hollow structures that can easily confuse the AI-CADx system. Noise perturbation experiments however circumvent this challenge.

As final proof, we qualitatively evaluate the spatial correlation of the malignancy heterogeneity identified by our CAM-based malignancy heat map with the surgical pathology. Due to not only the technical difficulty of automatically registering cross-modality images, i.e., digital histopathology images and ultrasound images, but also more importantly the retrospective nature of this study, we could not specifically collect the ultrasound images of the very nodular cross-sections that best corresponded to those of the histopathology images. Moreover, arbitrary shape changes can be introduced during the surgical operation and fully automatic registration between digital H&E histopathology images, and ultrasound images would also require training of a sufficient good segmentation model for cancer cell-rich regions in histopathology images, which is currently beyond our capacity of assessable resources. It is however important to note that the ultrasound feature evaluations of the highlighted regions performed by radiologists according to ACR TI-RADS criteria show a higher correlation with malignancy risks compared to the inactivated regions, demonstrating the malignancy relevance of the highlighted regions in our proposed variant of CAM heat map. This can be mainly attributed to two facts: the AI system has high accuracy in localizing thyroid nodules with a median dice coefficient >0.9, and the AI system has very high diagnostic accuracy with an AUC value of 0.9302 in this study for discriminating between malignant and benign nodules. The nodule segmentation is a crucial step for the computations of heat maps, and it proves that the corresponding area of the heat map is mainly in the lesion area of the nodule. Good performance in distinguishing malignant from benign nodules also plays a vital role in identifying the “hot” regions in the malignancy heat map to be the key regions for the diagnosis of malignant nodules. Furthermore, other recent studies have shown that the AI system has balanced specificity and sensitivity with overall diagnostic accuracy matching high-performing senior radiologists (24), can outperform senior radiologists in diagnosing rare thyroid carcinomas (25), and can be potentially helpful for discrimination between malignant and benign follicular-patterned thyroid lesions (21). Interestingly, the shapes and subregions of cancer cell-rich pathological images showed good correspondence to the heat maps computed from the ultrasound images, suggesting a promising potential to use the heat map visualization to guide targeted FNAB for more reliable sampling compared with conventional ultrasound-guided sampling.

Ultrasound-guided thyroid FNAB has been shown to improve the sampling accuracy for suspicious nodule identification (26). However, there is a trade-off between the reduction of patient discomfort that would put a constraint on the number of needle passes and the diagnostic accuracy that is limited by specimen adequacy. The CAM-based heat maps computed from AI-CADx systems with diagnostic performances comparable to or even better than those of senior radiologists (27–29) on ultrasound images with the capability of differentiating regional importance for malignancy diagnosis may provide additional guidance to localize diagnosis-enabling nodular regions, especially large ones, for more accurate sampling, given that the number of needle passes has to be limited. In addition, FNAB-based cytopathological examination is acknowledged to have a limitation in diagnosing follicular-patterned thyroid lesions (FPTLs) (30–32), while the AI system was found to be 69% accurate in differentiating thyroid follicular carcinoma from benign FPTL cases (21), suggesting that the heat maps developed on top of the AI system may provide better guidance than plain ultrasound for FPTL sampling by FNAB to help with newly developed proteomics-based diagnosis (33). Of course, for smaller thyroid nodules, it may be difficult to precisely guide FNAB of the segmented nodular regions based on the malignancy heat map. Meanwhile, it must be noticed that the FNAB selection of thyroid nodules has become increasingly conservative in clinical practice. FNAB for malignant suspicious thyroid nodules recommended by guidelines (23, 34) is commonly performed for nodules with the smallest diameter ≥10 mm. Therefore, it is of clinical significance for this study to guide FNAB of relatively large thyroid nodules with the heat maps. It may also be of special interest to investigate whether CAM-based heat maps on ultrasound images can be helpful for guiding core needle biopsies of, for instance, liver lesions, which was shown to have a complication rate of 10.6% for repeated biopsies with a diagnostic accuracy of 83.3% (35).

Furthermore, currently, the generation of CAMs is based on an AI-CADx system trained on static images. For real-world clinical applications, it will be beneficial to have such heat maps dynamically generated in real-time during ultrasound scanning, which would require the corresponding AI-CADx system to be able to operate in a dynamic mode with high diagnostic accuracy. Practically speaking, ultrasound reflections from needles might interfere with accurate heat map visualization in real time. Another noteworthy limitation of the proposed CAM and its variant presented in this study is that their visualization is currently limited to being two-dimensional and thus not very suited yet to visualizing the degree of malignancy suspiciousness of a targeted cross-sectional plane relative to that of the planes above and below. If a three-dimensional heat map visualization is to be developed such that additional guidance about how deep the needles shall be inserted into the nodules to acquire samplings can be possible. In addition to the applied basic CAM method, there are also other published variants such as Grad-CAM (36), Score-CAM (37), and Ablation-CAM (38). These methods of generating heat maps can in principle be investigated as well. However, it is for future studies to evaluate which heat map generation techniques can be the most useful for guiding FNAB. This work is mainly to show that CAM-based heat maps can visualize intra-nodular malignancy heterogeneity, and this may recommend better sites for FNAB sampling, which is to be evaluated in a separate study.

## Conclusion

The CAM and its variant generated on ultrasound images through a highly accurate AI-CADx system can provide differential importance of nodular regions for tumor malignancy prediction, which was validated by adding noise perturbations to different regions of thyroid nodules. Our proposed malignancy heat map offers quantitative visualization of malignancy heterogeneity within a tumor, and the highlighted regions are better correlated with the malignancy risk than the inactivated regions. The good spatial correspondence with post-operation pathology warrants clinical interests to investigate further whether such AI-based malignancy-heterogeneity visualization techniques can provide targeted guidance for needle-based aspiration biopsies of tumors in comparison with plain ultrasound imaging to improve sampling accuracy and reduce complications that may associate with the procedures.

## Data availability statement

The data analyzed in this study is subject to the following licenses/restrictions: It is not within the agreement with the participants to make the original dataset publicly available. The data that support the findings of this study are available on reasonable request from corresponding author L.W. after formal approval by the concerned Chinese regulating authorities.. Requests to access these datasets should be directed to wanglj844@zjcc.org.cn.

## Ethics statement

The studies involving human participants were reviewed and approved by The Cancer Hospital of the University of Chinese Academy of Sciences but waived in view of the retrospective nature of the study and all involved ultrasound image reviews being performed were part of the clinical routine. The patients/participants provided their written informed consent to participate in this study.

## Author contributions

Conceptualization: LPW and LX. Methodology: YW, JY, and WL. Software: YW, WL, and JY. Validation: LPW, LJW, and LX. Formal analysis: YW and DX. Investigation: LPW, YW, DX, and LJW. Resources: LJW and LX. Data curation: LPW, LJW, and DX. Writing—original draft preparation: YW and LX. Writing—review and editing: LX. Supervision: LJW and LX. Project administration: LJW and LX. Funding acquisition: DX and LPW. All authors have read and agreed to the published version of the manuscript.

## Supplementary material

Supplementary Figure 1 Influence of adding gradient sign noise perturbations to ultrasound images of different magnitudes b. (a and d) The original image of thyroid nodule and its surroundings. (b and e) The generated gradient sign noises with the respective magnitude of 0.0136 and 0.05. (c and f) The resulting images corresponding to noise perturbations given by panels b and e. (g) The measurement of how the diagnosis performances (by AUC values) of the AICADx system are influenced by noise perturbations with magnitudes b ranging from 0 to 0.1.

Supplementary Figure 2 The ROC curve of the AI-CADx system for thyroid nodule diagnosis and the corresponding AUC value.

Supplementary Figure 3 The diagnostic performance of the AI-CADx system directly on segmented regions above the binarization CAM threshold (represented as a ratio to the maximum temperature intensity 255).
