# Supplementary figures and images for: Differential regional importance mapping for thyroid nodule malignancy prediction with potential to improve needle aspiration biopsy sampling reliability
Source: Front Oncol. 2023 Apr 28;13:1136922. doi: 10.3389/fonc.2023.1136922 (PMC10175814; doi:10.3389/fonc.2023.1136922)

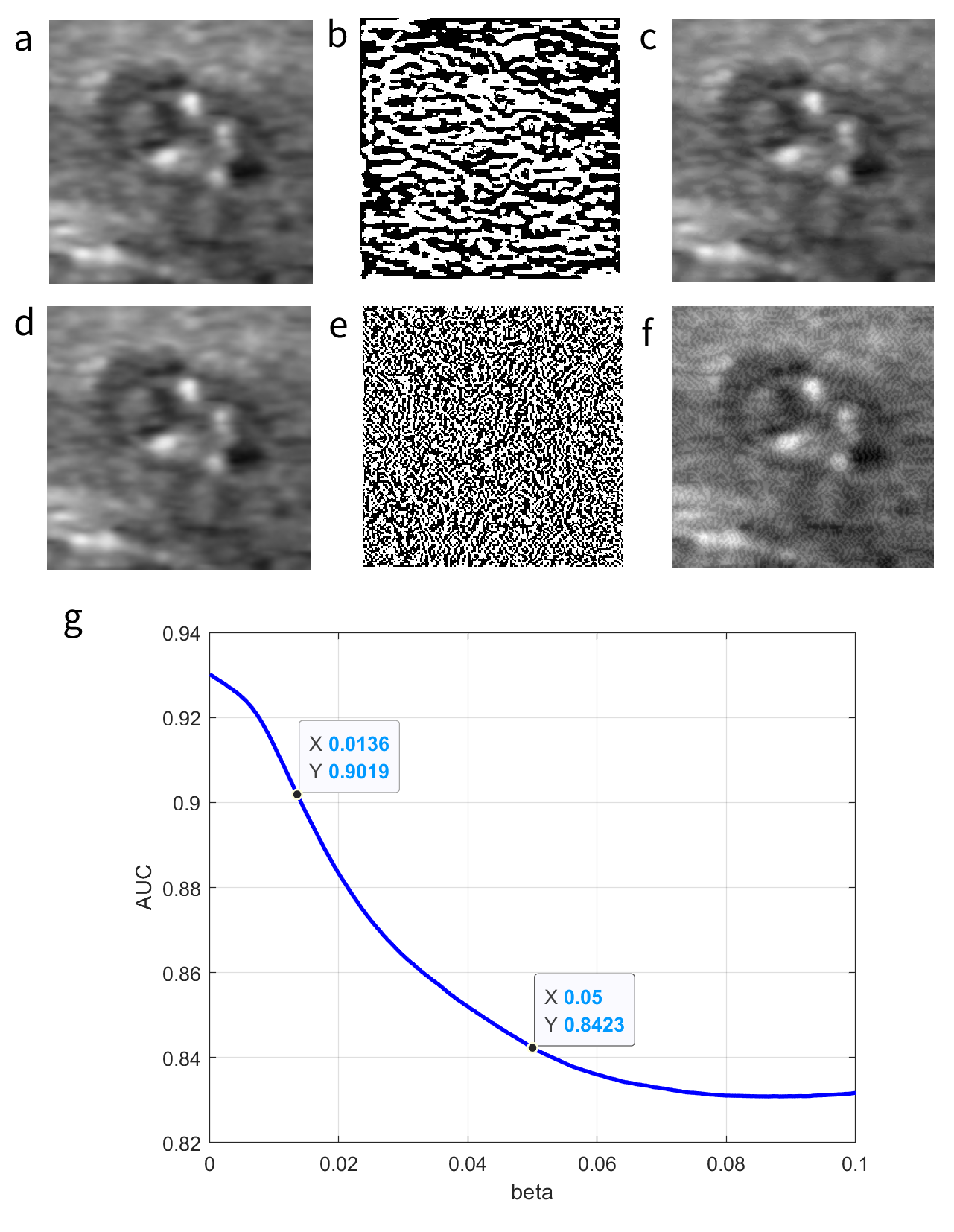

Supplement: Supplementary Figure 1 [file Image_1.png]

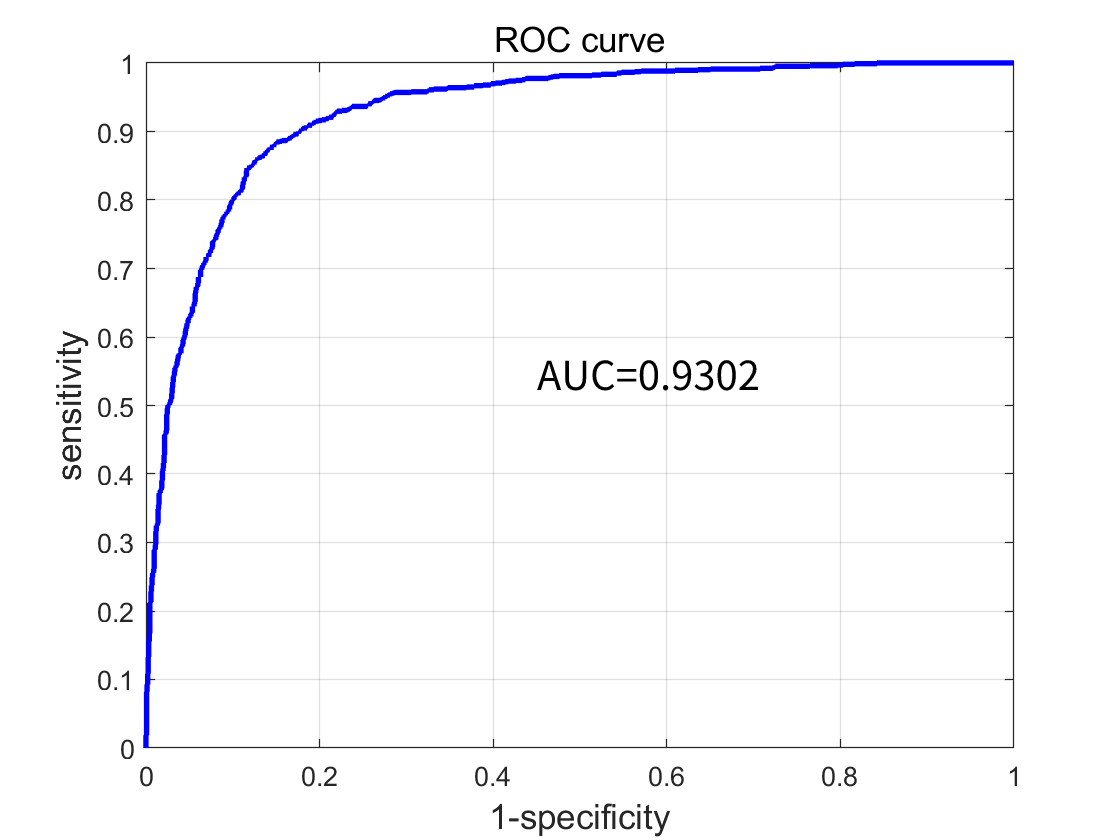

Supplement: Supplementary Figure 2 [file Image_2.png]

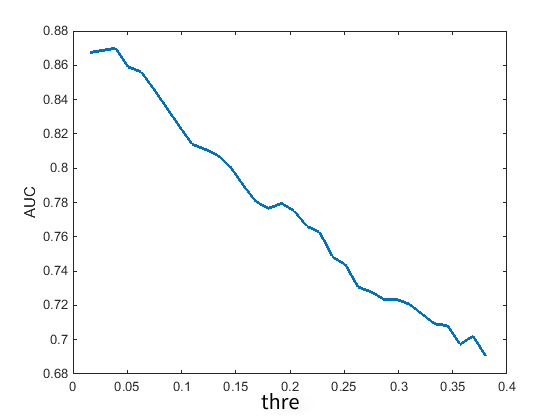

Supplement: Supplementary Figure 3 [file Image_3.png]
